# Immunogenicity, safety and consistency of seven lots of an inactivated COVID-19 vaccine in healthy children and adolescents: a randomized, double-blind, controlled, phase IV clinical trial

**DOI:** 10.3389/fimmu.2023.1320352

**Published:** 2024-01-05

**Authors:** Weijun Hu, Xiaoyu Liu, Xi Lu, Dan Zhang, Shuo Liu, Xianjin Gu, Dan Liu, Jianwen Sun, Tiantian Zhou, Xinge Li, Yongjun Gao, Yanwei Zhao, Guoliang Cui, Shaobai Zhang

**Affiliations:** ^1^ Institute of Immunization Program, Shaanxi Provincial Center for Disease Control and Prevention, Xi’an, China; ^2^ Medical Affairs Department, Sinovac Biotech Co., Ltd., Beijing, China; ^3^ Clinical Research and Development Center, Sinovac Biotech Co., Ltd., Beijing, China; ^4^ Department of Immunization Program, Yanliang District Center for Disease Control and Prevention, Xi’an, China; ^5^ Medical Affairs Department, Sinovac Life Sciences Co., Ltd., Beijing, China; ^6^ Quality Assurance Department, Sinovac Life Sciences Co., Ltd., Beijing, China

**Keywords:** CoronaVac, lot-to-lot consistency, immunogenicity, safety, children and adolescents

## Abstract

**Background:**

CoronaVac has been authorized worldwide for preventing coronavirus disease 2019. Information on the safety, immunogenicity and consistency of different lots and workshops of CoronaVac is presented here.

**Methods:**

In this randomized, double-blind, phase IV clinical trial in healthy children and adolescents aged 3-17 years, we aimed to assess the lot-to-lot and workshop-to-workshop consistency, as well as immunogenicity and safety of seven lots of commercial-scale CoronaVac from three workshops. Eligible participants were enrolled into three age cohorts (3-5, 6-11 and 12-17 years). Within each cohort, participants were randomly assigned to seven groups to receive two doses of CoronaVac, with four weeks apart. Serum samples were collected before the first dose and 28 days after the second dose for neutralizing antibody testing. The primary objective was to evaluate the consistency of immune response among different lots within workshop 2 or 3, as well as among different workshops. The primary endpoint was geometric mean titer (GMT) of neutralizing antibody at 28 days after full-course vaccination.

**Results:**

Between July 27^th^ and November 19^th^, 2021, a total of 2,520 eligible participants were enrolled. Results showed that 95% confidence intervals (CIs) of GMT ratios for all comparative groups among different lots or workshops were within the equivalence criteria of [0.67, 1.5]. The GMT and seroconversion rate for all participants were 126.42 (95%CI: 121.82, 131.19) and 99.86% (95%CI: 99.59%, 99.97%) at 28 days after two-dose vaccination. The incidences of adverse reactions were similar among seven lots, and most adverse reactions were mild in Grade 1, with no serious adverse event.

**Conclusion:**

CoronaVac is well-tolerated and can elicit a good immune response among children and adolescents. Lot-to-lot consistency results indicate stable manufacturing of commercial-scale CoronaVac.

## 1 Introduction

Coronavirus disease 2019 (COVID-19), caused by severe acute respiratory syndrome coronavirus 2 (SARS-CoV-2), is a highly contagious respiratory disease that has radically impacted the world since its emergence in December 2019 (1). COVID-19 symptoms can vary from mild to severe, with common symptoms of fever, cough, sore throat, and shortness of breath (2). While most individuals may experience mild to moderate symptoms or even be asymptomatic after infection, vulnerable populations, such as the elderly and individuals with pre-existing health conditions, are at a high risk of developing severe illnesses that require medical assistance or hospitalization (3, 4). Given its significant toll on public health, economies, and societies, the World Health Organization (WHO) designated COVID-19 as a public health emergency of international concern on January 30, 2020, and subsequently declared it a pandemic on March 11, 2020 (5). To contain and mitigate the transmission and impact of COVID-19, many countries have implemented various preventive measures, including lockdown, wearing mask, social distancing, and vaccination campaign, of which vaccination is usually treated as the most effective measure (6, 7).

The development of COVID-19 vaccine has been a top priority since the beginning of the pandemic, leading to an expedited timeline for vaccine development. Scientists and biopharmaceutical companies around the world have been working relentlessly to develop safe and effective vaccines to prevent COVID-19 (8). CoronaVac, an inactivated COVID-19 vaccine, was developed by Sinovac Life Sciences Co., Ltd. (hereafter referred to as Sinovac) beginning in early 2020. Extensive clinical trials were conducted by Sinovac to assess the safety and efficacy of CoronaVac, involving tens of thousands of participants in multiple countries, including China, Brazil, Turkey, Indonesia, and Chile. Results of phase I to III clinical trials in adults aged ≥18 years showed that CoronaVac was safe and could induce a protective immune response, with efficacy against symptomatic COVID-19 ranging from 50.7% to 83.5% (9–11). According to these promising results in adults, CoronaVac was granted conditional market approval by the National Medical Products Administration (NMPA) of China on 5 February 2021, and was validated by WHO for emergency use on 1 June 2021. In addition, based on well-performed safety and immunogenicity data (12), CoronaVac was then approved by NMPA for emergency use in children and adolescent aged 3-17 years on 28 May 2021. In August 2022, the Health Bureau of Hong Kong, China and Chilean Public Health Institute firstly approved CoronaVac on children aged 6 months and older. CoronaVac is now being used widely around the world in a large-scale population.

As required by NMPA, lot-to-lot consistency studies were recommended to be carried out to provide stability data of commercial-scale CoronaVac. A previous phase IV clinical trial in health adults aged 26-45 years showed lot-to-lot consistency of three lots of CoronaVac on a commercial scale (13), using the vaccines produced in workshop 1 that has been certificated by WHO since 2021. Sinovac further expanded two workshops (workshop 2 and 3) to fulfill the planned production capacity and meet the shortage of COVID-19 vaccine worldwide. Hence, it is essential to assess the stability and consistency of the two newly established workshops in comparison to the validated workshop 1. In addition, CoronaVac has also been authorized for children and adolescent, and stable manufacturing evidence on this population is still unclear. Therefore, the aim of this trial was to assess the lot-to-lot consistency, immunogenicity and safety of seven lots of commercial-scale CoronaVac from three different workshops, among healthy children and adolescents aged 3-17 years.

## 2 Materials and methods

### 2.1 Study design and participants

This study was a single-center, randomized, double-blind, phase IV clinical trial, which was conducted in Yanliang District, Xian City, Shaanxi Province, China, from July 27^th^ to November 19^th^, 2021. Healthy children and adolescents aged 3-17 years, whose legal guardians and themselves were willing to sign the written informed consent and provide the legal identification, were eligible for enrolment. The main exclusion criteria for the first dose included (1) having a history of SARS-CoV-2 infection or COVID-19 by inquiring the medical history; (2) previously receiving any COVID-19 vaccine; (3) having a history of asthma, allergic to vaccines or vaccine ingredients, or having serious adverse reactions to vaccines, such as urticaria, dyspnea, angioneurotic edema; (4) suffering from autoimmune diseases (such as systemic lupus erythematosus) or being in an immunodeficient/immunosuppressed state (such as HIV/AIDS, post-organ transplantation); (5) with severe neurological disease (epilepsy, convulsions or convulsions) or mental illness; (6) receipt of blood products within 3 months prior to receiving the investigational vaccine, or planning to receive the above treatments during the study period; (7) receipt of other investigational vaccine or drugs in the past 30 days; (8) receipt live attenuated vaccines in the past 14 days or inactivated or subunit vaccines in the past 7 days; (9) having axillary temperature more than 37.0°C at the time of screening; (10) having any other factors that are not suitable for participating in the clinical trial according to the investigators’ judgment. The main exclusion criteria for the second dose included (1) receiving other COVID-19 vaccine before dose 2; (2) getting SARS-CoV-2 infection before dose 2 by inquiring the medical history; (3) having serious adverse reaction related to study vaccine; (4) having axillary temperature more than 37.0°C at the time of second vaccination; (5) having any other factors that are not suitable for vaccination according to the investigators’ judgment.

This trial was approved by the ethics committee of Shaanxi Provincial Centre for Disease Control and Prevention. Written informed consents were obtained before screening. For participants aged 8 years or older, both their legal guardians and participants were required to sign the informed consent forms, while only their legal guardians signed for participants below eight years. This trial was registered in the ClinicalTrials.gov database with an identifier of NCT05112913. This trial was conducted in accordance with the principles of the International Council for Harmonization Good Clinical Practice, the Declaration of Helsinki and Chinese regulatory requirements.

### 2.2 Randomization and blinding

Eligible participants were enrolled into three different age cohorts (3-5, 6-11 and 12-17 years old). Within each cohort, participants were randomly assigned, in a ratio of 1:1:1:1:1:1:1, to receive two doses of CoronaVac from one of the seven lots from three workshops on a commercial scale, with four weeks apart. Randomization codes were generated by the statistician using the method of age-stratified block randomization, and were then allocated to each participant in the sequence of enrolment order. Participants received the vaccines labelled with the same code number. Investigators, participants, and laboratory staff were all blinded to the vaccine lot allocation.

The randomized statistician and other blind coding personnel, who were not allowed to participate in other works of this trial, were responsible for blinding the vaccines. The blind code file was then sealed and maintained by the randomized statistician and was not to be opened until the unblinding procedure of the study.

### 2.3 Study vaccines

The manufacturing process and facilities for CoronaVac have been described previously (12, 14, 15). In brief, CoronaVac was an inactivated SARS-CoV-2 whole virion vaccine with an adjuvant of aluminum hydroxide. CoronaVac was manufactured by SARS-CoV-2 CZ02 strain, which was inoculated in African green monkey kidney cells (Vero cells) and then was harvested, inactivated using β-propiolactone, concentrated, purified, and adsorbed onto aluminum hydroxide. The aluminum hydroxide complex was then diluted in a sodium chloride, phosphate-buffered saline, and water solution before being sterilized and filtered ready for injection. CoronaVac was prepared in a Good Manufacturing Practice-accredited facility of Sinovac, which was periodically inspected by NMPA committee for compliance.

CoronaVac used in this trial was packed in prefilled syringes and stored at 2-8°C. It was administered intramuscularly into the lateral deltoid muscle of upper arm, at a dose of 600SU/0.5ml (3μg antigen). In this trial, the schedule of vaccination was two doses on Day 0,28. Seven lots of CoronaVac from three workshops, on a commercial scale, were used in this trial. Workshop 1 provided one lot of CoronaVac (lot number: D202104004), and workshop 2 and 3 each provided three consecutive lots (lot number of D202105014, U202106002 and A202106042 for workshop 2, and A202105034, A202106035 and A202106036 for workshop 3, respectively). All lots of CoronaVac had been approved by the National Institutes for Food and Drug Control (NIFDC) of China.

### 2.4 Immunogenicity assessment

For all participants, each time 3 ml of blood samples were collected before the first dose vaccination (Day 0) and at 28 days after the second dose vaccination (Day 56). Neutralizing antibody titers against the ancestral strain were then tested using a micro-cytopathogenic effect assay, which was done by Sinovac (12). Neutralizing antibody titers were presented as half of the lower limit of quantitation (LLOQ) if they were lower than LLOQ (1:4). Seropositive refers to titer ≥1:8. Seroconversion refers to titer changes from <1:8 to ≥1:8 after vaccination after full course of vaccination, or at least 4-fold increases if baseline titer is ≥1:8.

The immunogenicity objectives were to evaluate the consistency of neutralizing antibody response among different lots within workshop 2 or 3, as well as the consistency between different workshops. The primary endpoint was geometric mean titer (GMT) of neutralizing antibody at 28 days after full course of vaccination. The second endpoints included seropositive rate, seroconversion rate, and geometric mean increase (GMI) of neutralizing antibody. The immunogenicity assessment was conducted in the per-protocol set (PPS), which included all participants who completed two doses of vaccination, had available neutralizing antibody results above and complied with the protocol. The time windows for Day 28 and Day 56 were +10 days.

### 2.5 Safety assessment

Participants stayed in the trial center for at least 30 minutes after each vaccination for immediate adverse events (AE) observation. Within28 days after each vaccination, participants and their legal guardians were required to record any local AEs (at the injection site) and systemic AEs on the electronic diary cards through an application program using their phones. At 7 and 28 days after each vaccination, investigators verified the AE recordings via telephone or face-to-face interview with participants and their legal guardians. In this trial, solicited period referred to the first 7 days after vaccination, and 8 to 28 days was then defined as the unsolicited period. Solicited local adverse events included pain, induration, swelling, erythema, rash, and pruritus, which occurred at the injection site in the solicited period. Solicited systemic adverse events included fever, acute hypersensitive reaction, mucocutaneous disorder, diarrhea, decreased appetite, vomiting, nausea, myalgia, headache, cough, and fatigue, which occurred in the solicited period. Serious adverse events (SAE) and adverse events of special interest (AESI) were recorded throughout the trial period up to 28 days after full course of vaccination. The reported AEs were graded according to the NMPA guideline (16). The causal relationship between AE and vaccination was determined by the investigators.

The safety endpoints in this trial included the incidence of solicited local and systemic adverse reactions and unsolicited adverse reactions, as well as SAE and AESI until 28 days after full course of vaccination (Day 0-56). Safety assessment was conducted in the safety set (SS) of participants who received at least one dose of vaccination.

### 2.6 Sample size determination and statistical analysis

For the consistency assessment, equivalence is considered to be fulfilled when the 95% confidence interval (CI) for the inter-group GMT ratio were between 0.67 and 1.5, which was equivalent to the GMT difference after log_10_ transformation between -0.176 and 0.176. The immunogenicity endpoints were decided to be assessed in 2,520 enrolled participants, of which 360 participants were in each lot with 52, 154 and 154 participants aged 3-5, 6-11 and 12-17 years, respectively. This sample size would allow for a statistical power of 90% (1-β) to detect equivalence of either two groups, with an expected drop-out rate of 15% and a two-sided significance level of 5% (α).

Generally, Pearson’s chi-squared test, Fisher’s exact test or one-way ordered Cochran–Mantel–Haenszel (CMH) test were applied to analyze categorical variables for group comparison, and analysis of variance (ANOVA) were used for continuous variables to compare the difference between groups. Observed GMTs and corresponding 95%CIs were calculated based on a standard normal distribution of logarithmic transformed GMT. The 95%CIs of seropositive rate and seroconversion rate were calculated using Clopper-Pearson analysis. For equivalence analysis, analysis of covariance (ANCOVA) model was applied, where the logarithmic transformed GMT 28 days after full course of vaccination was the dependent variable, vaccines groups (lots or workshops) were the fixed effects, and logarithmic transformed GMT before vaccination was the covariate. Adjusted GMT ratios and corresponding 95%CIs were then estimated by the geometric least-square mean from the ANCOVA model for the comparisons between each pair of groups (Lot 1/Lot 2, Lot 1/Lot 3, Lot 2/Lot 3, Workshop 1/Workshop 2, Workshop 1/Workshop 3, and Workshop 2/Workshop 3). When both sides of 95%CIs of GMT ratios fell into [0.67, 1.5], the consistency of the comparison groups was fulfilled.

Subgroup analyses were also performed by age groups (3-5, 6-11 and 12-17 years) for neutralizing antibodies and incidence of adverse reactions. Exploratory analyses were applied to evaluate the antibodies response at 7 and 14 days after vaccination in the immunogenicity subgroup participants. Hypothesis testing was two-sided, and p values of less than 0.05 were considered to be significant. All analyses were conducted with SAS (Version 9.4, SAS Institute Inc., Cary, USA).

## 3 Results

### 3.1 Study participants characteristics

**Figure 1:**
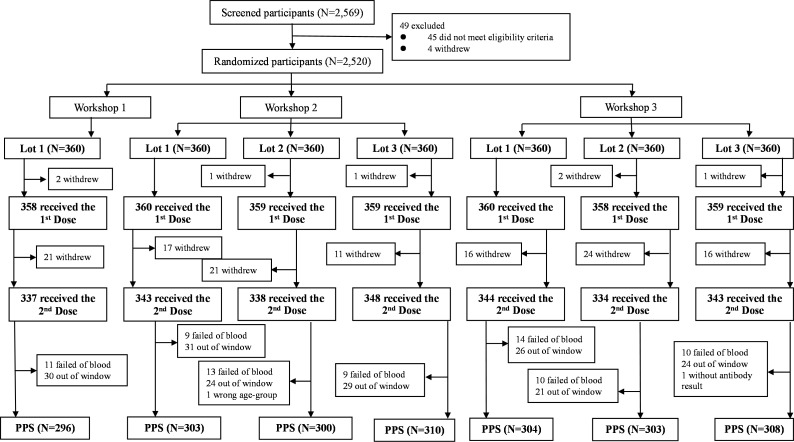
Flow chart of study profile.

Between July 27^th^ and November 19^th^, 2021, a total of 2,569 participants were screened and 49 out of them did not meet the eligibility criteria or withdrew, resulting in 2,520 eligible participants enrolled (**Figure 1**). Given that seven participants withdrew with no reason before the first dose of vaccination, 2,513 participants were finally included in the SS, of whom 389 participants were not included in the PPS, mainly due to withdrawal, failed of blood sampling, and out-of-window visit. Detailed reasons were shown in **Figure 1**. Finally, 2,124 participants (84.29%) were included in PPS, of which 296 were included in lot 1 of workshop 1, 303, 300 and 310 were included in lot 1, 2 and 3 of workshop 2, as well as 304, 303 and 308 were included in lot 1, 2 and 3 of workshop 3, respectively (**Figure 1**).

The demographic characteristics of enrolled participants were similar between different lots or workshops either in SS or PPS, with no statistically significant difference in age, gender, ethnicity, height, and weight (**Table 1**). In specific, among 2,513 participants in SS, the average age was 10.3 years, 51.33% were male, and most participants (98.97%) were Han ethnic. For the three workshops, the average ages were 10.3, 10.3, and 10.3 years, the percentages of male were 51.96%, 49.63%, and 52.83%, and Han ethnic accounted for 98.60%, 98.79%, and 99.26%, respectively. In SS, 361 (14.37%), 1,075 (42.78%), and 1,077 (42.86%) participants were in the age cohorts of 3-5, 6-11 and 12-17 years old, respectively, and the proportions of the three age groups were similar among the three workshops. Among 2,124 participants in PPS, the average age was 10.4 years, 51.41% were male, and most participants (98.87%) were Han ethnic. For the three workshops, the average ages were 10.5, 10.4, and 10.4 years, the percentages of male were 51.69%, 50.60%, and 52.13%, and Han ethnic accounted for 98.31%, 98.69%, and 99.23%, respectively. In PPS, 290 (13.65%), 887 (41.76%), and 947 (44.59%) participants were in the age cohorts of 3-5, 6-11 and 12-17 years old, respectively, and the proportions of the three age groups were similar among the three workshops.

**Table 1:** Demographic characteristics of study participants (SS and PPS).

	Workshop 1	Workshop 2					Workshop 3					Total	P (3 workshops)	P (7 lots)
	Lot 1	Lot 1	Lot 2	Lot 3	Sub-total	P	Lot 1	Lot 2	Lot 3	Sub-total	P			
**SS, N**	**358**	**360**	**359**	**359**	**1,078**		**360**	**358**	**359**	**1,077**		**2,513**		
Age, year														
Mean (SD)	10.3(3.87)	10.3(3.80)	10.3(3.83)	10.3(3.82)	10.3(3.82)	0.9893	10.3(3.84)	10.4(3.76)	10.3(3.81)	10.3(3.80)	0.9758	10.3(3.81)	0.9604	0.8537
3-5 y, n (%)	52(14.53)	52(14.44)	50(13.93)	52(14.48)	154(14.29)	0.9895	52(14.44)	51(14.25)	52(14.48)	155(14.39)	0.9965	361(14.37)	0.9996	1.0000
6-11 y, n (%)	152(42.46)	154(42.78)	155(43.18)	154(42.90)	463(42.95)		154(42.78)	153(42.74)	153(42.62)	460(42.71)		1,075(42.78)		
12-17 y, n (%)	154(43.02)	154(42.78)	154(42.90)	153(42.62)	461(42.76)		154(42.78)	154(43.02)	154(42.90)	462(42.90)		1,077(42.86)		
Male n (%)	186(51.96)	170(47.22)	177(49.30)	188(52.37)	535(49.63)	0.3817	199(55.28)	194(54.19)	176(49.03)	569(52.83)	0.2002	1,290(51.33)	0.3203	0.2843
Han ethnic n (%)	353(98.60)	357(99.17)	353(98.33)	355(98.89)	1,065(98.79)	0.5777	356(98.89)	356(99.44)	357(99.44)	1,069(99.26)	0.6081	2,487(98.97)	0.4354	0.7231
Height, cm														
Mean (SD)	145(22.37)	145(21.85)	145(21.74)	145(22.19)	145(21.91)	0.9691	145(22.90)	146(21.39)	145(21.90)	145(22.05)	0.9800	145(22.03)	0.9339	0.7706
Weight, kg														
Mean (SD)	41.6(17.07)	41.7(17.97)	40.8(16.47)	41.0(15.77)	41.2(16.75)	0.7409	42.1(17.03)	42.0(16.71)	41.6(17.01)	41.9(16.90)	0.9176	41.5(16.86)	0.6447	0.5455
**PPS, N**	**296**	**303**	**300**	**310**	**913**		**304**	**303**	**308**	**915**		**2124**		
Age, year														
Mean (SD)	10.5(3.78)	10.4(3.68)	10.4(3.78)	10.4(3.86)	10.4(3.77)	0.9618	10.4(3.79)	10.4(3.68)	10.4(3.72)	10.4(3.73)	0.9875	10.4(3.75)	0.9394	0.8400
3-5 y, n (%)	40(13.51)	39(12.87)	40(13.33)	46(14.84)	125(13.69)	0.9319	44(14.47)	41(13.53)	40(12.99)	125(13.66)	0.9774	290(13.65)	0.9396	1.0000
6-11 y, n (%)	121(40.88)	128(42.24)	130(43.33)	123(39.68)	381(41.73)		123(40.46)	130(42.90)	132(42.86)	385(42.08)		887(41.76)		
12-17 y, n (%)	135(45.61)	136(44.88)	130(43.33)	141(45.48)	407(44.58)		137(45.07)	132(43.56)	136(44.16)	405(44.26)		947(44.59)		
Male n (%)	153(51.69)	149(49.17)	153(51.00)	160(51.61)	462(50.60)	0.8218	164(53.95)	166(54.79)	147(47.73)	477(52.13)	0.1611	1,092(51.41)	0.8033	0.6122
Han ethnic n (%)	291(98.31)	300(99.01)	294(98.00)	307(99.03)	901(98.69)	0.4448	301(99.01)	301(99.34)	306(99.35)	908(99.23)	0.8627	2,100(98.87)	0.3333	0.6390
Height, cm														
Mean (SD)	147(22.35)	146(21.72)	146(22.07)	146(22.55)	146(22.10)	0.9370	146(23.28)	146(21.58)	146(21.20)	146(22.01)	0.9978	146(22.09)	0.9456	0.8033
Weight, kg														
Mean (SD)	42.3(17.46)	42.8(18.53)	41.7(16.76)	41.7(15.98)	42.0(17.11)	0.6516	42.9(17.41)	42.5(17.01)	41.9(17.31)	42.4(17.23)	0.7588	42.2(17.20)	0.8782	0.7690

Chi-square test for sex and ethnic; analysis of variance (ANOVA) for age, height and weight; One-way ordered CMH chi-square test for categorical age.

### 3.2 Immunogenicity

The immunogenicity results in PPS are shown in **Table 2**. Except for one participant in lot 1 of workshop 1 and two participants in lot 3 of workshop 2, participants were seronegative (neutralizing antibody titers <1:8) for neutralizing antibody before vaccination, with a GMT value of 2.06 (95%CI: 2.04, 2.07) for all participants. The observed GMT for all participants was 126.42 (95%CI: 121.82, 131.19) at 28 days after two-dose vaccination, ranging from 118.78 to 134.97 among seven lots. The seroconversion rate for all participants was 99.86% (95%CI: 99.59%, 99.97%) at 28 days after two-dose vaccination, ranging from 99.32% to 100% among seven lots. The observed GMI for all participants was 61.49 (95%CI: 59.21, 63.85) at 28 days after two-dose vaccination, ranging from 57.89 to 65.64 among seven lots. As for the three workshops, the observed GMTs at 28 days after two-dose vaccination were 122.33 (95%CI: 110.53, 135.38), 128.23 (95%CI: 121.12, 135.75), and 125.96 (95%CI: 119.13, 133.19) for workshop 1, 2 and 3, respectively. The seroconversion rates at 28 days after two-dose vaccination were 99.32% (95%CI: 97.58%, 99.92%), 100.00% (95%CI: 99.60%, 100.00%), and 99.89% (95%CI: 99.39%, 100.00%) for workshop 1, 2 and 3, respectively. The observed GMIs at 28 days after two-dose vaccination were 59.75 (95%CI: 53.94, 66.18), 62.33 (95%CI: 58.80, 66.08), and 61.22 (95%CI: 57.86, 64.79) for workshop 1, 2 and 3, respectively. Overall, no statistically significant difference was observed in all immunogenicity endpoints before and after full course of vaccination among the seven lots or the three workshops (**Table 2**).

**Table 2:** Immunogenicity of neutralizing antibody before and 28 days after two-dose vaccination (PPS).

	Workshop 1	Workshop 2					Workshop 3					Total (N=2,124)	P value (3 workshops)	P value (7 lots)
	Lot 1 (N=296)	Lot 1 (N=303)	Lot 2 (N=300)	Lot 3 (N=310)	Sub-total (N=913)	P	Lot 1 (N=304)	Lot 2 (N=303)	Lot 3 (N=308)	Sub-total (N=915)	P			
Before vaccination														
Seropositive														
n (%)	1(0.34)	0(0.00)	0(0.00)	2(0.65)	2(0.22)	0.3327	0(0.00)	0(0.00)	0(0.00)	0(0.00)	NA	3(0.14)	0.1300	0.2607
95%CI	(0.01, 1.87)	NA	NA	(0.08, 2.31)	(0.03, 0.79)		NA	NA	NA	NA		(0.03, 0.41)		
**GMT,** 95%CI	2.05 (2.02, 2.08)	2.05 (2.02, 2.08)	2.06 (2.02, 2.09)	2.06 (2.03, 2.10)	2.06 (2.04, 2.08)	0.8886	2.05 (2.02, 2.09)	2.05 (2.02, 2.08)	2.07 (2.03, 2.11)	2.06 (2.04, 2.08)	0.7591	2.06 (2.04, 2.07)	0.8677	0.9826
At 28 days after vaccination														
Seropositive														
n (%)	294(99.32)	303(100.00)	300(100.00)	310(100.00)	913(100.00)	NA	304(100.00)	303(100.00)	307(99.68)	914(99.89)	1.0000	2,121(99.86)	0.0527	0.0701
95%CI	(97.58, 99.92)	(98.79, 100.00)	(98.78, 100.00)	(98.82, 100.00)	(99.60, 100.00)		(98.79, 100.00)	(98.79, 100.00)	(98.20, 99.99)	(99.39, 100.0)		(99.59, 99.97)		
Seroconversion														
n (%)	294(99.32)	303(100.00)	300(100.00)	310(100.00)	913(100.00)	NA	304(100.00)	303(100.00)	307(99.68)	914(99.89)	1.0000	2,121(99.86)	0.0527	0.0701
95%CI	(97.58, 99.92)	(98.79, 100.00)	(98.78, 100.00)	(98.82, 100.00)	(99.60, 100.00)		(98.79, 100.00)	(98.79, 100.00)	(98.20, 99.99)	(99.39, 100.00)		(99.59, 99.97)		
**GMT,** 95%CI	122.33 (110.53, 135.38)	118.78 (107.40, 131.37)	134.97 (122.86, 148.27)	131.51 (118.80, 145.58)	128.23 (121.12, 135.75)	0.1670	123.60 (111.82, 136.61)	126.57 (115.31, 138.93)	127.74 (115.86, 140.83)	125.96 (119.13, 133.19)	0.8878	126.42 (121.82, 131.19)	0.7116	0.6032
**GMI,** 95%CI	59.75 (53.94, 66.18)	57.89 (52.24, 64.15)	65.64 (59.66, 72.22)	63.73 (57.37, 70.79)	62.33 (58.80, 66.08)	0.1991	60.19 (54.39, 66.60)	61.71 (56.18, 67.79)	61.78 (55.92, 68.25)	61.22 (57.86, 64.79)	0.9164	61.49 (59.21, 63.85)	0.7608	0.6723
Adjusted GMT Ratio (95%CI)*														
Lot 1/Lot 2*		0.88(0.76, 1.01)	0.88(0.76, 1.01)				0.98(0.85, 1.12)	0.98(0.85, 1.12)						
Lot 1/Lot 3		0.90(0.78, 1.04)		0.90(0.78, 1.04)			0.97(0.84, 1.11)		0.97(0.84, 1.11)					
Lot 2/Lot 3			1.02(0.89, 1.18)	1.02(0.89, 1.18)				0.99(0.86, 1.14)	0.99(0.86, 1.14)					
Workshop 1/Workshop 2	0.95(0.85, 1.07)				0.95(0.85, 1.07)									
Workshop 1/Workshop 3	0.97(0.87, 1.09)									0.97(0.87, 1.09)				
Workshop 2/Workshop 3					1.02(0.94, 1.10)					1.02(0.94, 1.10)				

Seropositive indicates the GMT level ≥1:8. Seroconversion indicates the GMT changes from <1:8 to ≥1:8 after vaccination, or at least 4-fold increase after vaccination if baseline GMT is ≥1:8.

* Adjusted GMT ratios were estimated by the geometric least-square mean estimates based on analysis of covariance (ANCOVA) model.

In subgroup analyses by age, we found an increased trend of immune response with age decreasing, where the highest GMT at 28 days after two-dose vaccination was observed in participants aged 3-5 years [240.86 (95%CI: 222.78, 260.42)], followed by participants aged 6-11 years [155.12 (95%CI: 147.88, 162.71)] and 12-17 years [85.67 (95%CI: 81.06, 90.55)] (**Table S1**). The seroconversion rates at 28 days after two-dose vaccination were 100.00% (95%CI: 98.74%, 100.00%), 100.00% (95%CI: 99.58%, 100.00%), and 99.68% (95%CI: 99.08%, 99.93%) for participants aged 3-5 years, 6-11 years, and 12-17 years, respectively, with no statistically significant difference observed (**Table S1**).

### 3.3 Lot-to-lot consistency

For lot-to-lot consistency analyses, as shown in **Table 2**, the adjusted GMT ratios of neutralizing antibody between lot 1 and 2, lot 1 and 3, and lot 2 and 3 in workshop 2 were 0.88 (95%CI: 0.76, 1.01), 0.90 (95%CI: 0.78, 1.04), and 1.02 (95%CI: 0.89, 1.08), respectively. The adjusted GMT ratios of neutralizing antibody between lot 1 and 2, lot 1 and 3, and lot 2 and 3 in workshop 3 were 0.98 (95%CI: 0.85, 1.12), 0.97 (95%CI: 0.84, 1.11), and 0.99 (95%CI: 0.86, 1.14), respectively. As for the workshops, the adjusted GMT ratios were 0.95 (95%CI: 0.85, 1.07), 0.97 (95%CI: 0.87, 1.09), and 1.02 (95%CI: 0.94, 1.10) between workshop 1 and 2, workshop 1 and 3, and workshop 2 and 3, respectively. Finally, for all comparative groups (lots or workshops), both sides of 95%CIs of GMT ratios were within [0.67, 1.5] and the equivalence criteria were fulfilled (**Table 2**). In addition, the highest and lowest GMT values after full course of vaccination were observed in lot 1 (GMT=134.97) and lot 2 (GMT=118.78) of workshop 2, where the GMT ratio also met the equivalence criteria between these two lots (**Table 2**).

### 3.4 Safety

**Figure 2:**
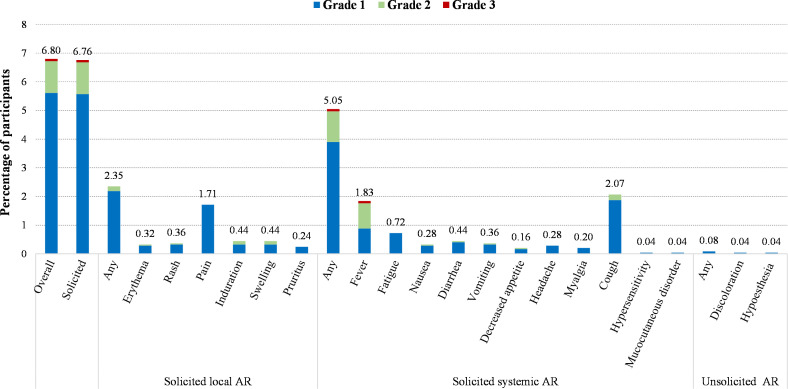
Incidence of adverse reactions (AR) after two-dose vaccination according to the grade of serivity (SS, 0-56 days).

A total of 171 out of 2,513 participants (6.80%) in SS reported adverse reactions after two-dose vaccination during Day 0-56, and the most common one was solicited adverse reactions (6.76%) (**Figure 2** and **Table 3**). The incidence of solicited local adverse reactions was 2.35%, of which injection-site pain was the most reported (1.71%), followed by induration (0.44%), swelling (0.44%), rash (0.36%), erythema (0.32%), and pruritus (0.24%). The overall incidence of solicited systemic adverse reactions was 5.05%, of which cough was the most reported (2.07%), followed by fever (1.83%), fatigue (0.72%), diarrhea (0.44%), and so on (**Figure 2**). In addition, the overall incidences of adverse reactions among seven lots ranged from 5.03% in lot 2 of workshop 3 to 9.19% in lot 3 of workshop 2, with no statistically significant difference. The incidences of all specific adverse reactions were also similar among seven lots (**Table 3**). As shown in **Figure 1**, most of adverse reactions were mild in grade 1, with a proportion of 82.46% (141/171). Two adverse reactions in grade 3 were observed, which were solicited adverse reaction fever. No adverse reaction was in grade 4, and no SAE or AESI were observed during the study period. **Table S2** shows the results of comparing the safety data after the first dose with that after the second dose. The overall incidence of solicited adverse reactions was 5.69% (143/2,513) within 28 days after the first dose, which was significantly higher than that (1.68%, 40/2,387) after the second dose (**Table S2**).

**Table 3:** Incidence of adverse reactions (AR) after two-dose vaccination among different lots (SS, 0-56 days).

	Workshop 1	Workshop 2			Workshop 3			P
	Lot 1 (N=358)	Lot 1 (N=360)	Lot 2 (N=359)	Lot 3 (N=359)	Lot 1 (N=360)	Lot 2 (N=358)	Lot 3 (N=359)	
**Overall AR**	**24(6.70%)**	**22(6.11%)**	**25(6.96%)**	**33(9.19%)**	**26(7.22%)**	**18(5.03%)**	**23(6.41%)**	**0.4826**
**Solicited AR**	**24(6.70%)**	**22(6.11%)**	**25(6.96%)**	**32(8.91%)**	**26(7.22%)**	**18(5.03%)**	**23(6.41%)**	**0.5695**
Local AR	11(3.07%)	7(1.94%)	7(1.95%)	9(2.51%)	9(2.50%)	8(2.23%)	8(2.23%)	0.9632
Erythema	0(0.00%)	2(0.56%)	2(0.56%)	0(0.00%)	1(0.28%)	1(0.28%)	2(0.56%)	0.6424
Rash	3(0.84%)	0(0.00%)	2(0.56%)	0(0.00%)	2(0.56%)	1(0.28%)	1(0.28%)	0.4446
Pain	7(1.96%)	4(1.11%)	4(1.11%)	8(2.23%)	7(1.94%)	7(1.96%)	6(1.67%)	0.8711
Induration	1(0.28%)	3(0.83%)	1(0.28%)	1(0.28%)	2(0.56%)	0(0.00%)	3(0.84%)	0.5555
Swelling	1(0.28%)	3(0.83%)	1(0.28%)	1(0.28%)	2(0.56%)	0(0.00%)	3(0.84%)	0.5555
Pruritus	1(0.28%)	0(0.00%)	0(0.00%)	3(0.84%)	0(0.00%)	1(0.28%)	1(0.28%)	0.2362
Systemic AR	17(4.75%)	17(4.72%)	19(5.29%)	27(7.52%)	18(5.00%)	11(3.07%)	18(5.01%)	0.2625
Fever	6(1.68%)	8(2.22%)	7(1.95%)	8(2.23%)	8(2.22%)	5(1.40%)	4(1.11%)	0.8787
Fatigue	2(0.56%)	2(0.56%)	3(0.84%)	5(1.39%)	1(0.28%)	0(0.00%)	5(1.39%)	0.2034
Nausea	1(0.28%)	0(0.00%)	1(0.28%)	0(0.00%)	1(0.28%)	1(0.28%)	3(0.84%)	0.4210
Diarrhea	2(0.56%)	1(0.28%)	4(1.11%)	2(0.56%)	1(0.28%)	0(0.00%)	1(0.28%)	0.3998
Vomiting	1(0.28%)	0(0.00%)	1(0.28%)	2(0.56%)	2(0.56%)	1(0.28%)	2(0.56%)	0.8488
Decreased appetite	0(0.00%)	1(0.28%)	2(0.56%)	0(0.00%)	1(0.28%)	0(0.00%)	0(0.00%)	0.3695
Headache	1(0.28%)	2(0.56%)	0(0.00%)	0(0.00%)	1(0.28%)	2(0.56%)	1(0.28%)	0.6752
Myalgia	0(0.00%)	2(0.56%)	2(0.56%)	0(0.00%)	0(0.00%)	0(0.00%)	1(0.28%)	0.2689
Cough	7(1.96%)	6(1.67%)	8(2.23%)	11(3.06%)	8(2.22%)	6(1.68%)	6(1.67%)	0.8449
Hypersensitivity	1(0.28%)	0(0.00%)	0(0.00%)	0(0.00%)	0(0.00%)	0(0.00%)	0(0.00%)	0.4207
Mucocutaneous disorder	0(0.00%)	0(0.00%)	0(0.00%)	0(0.00%)	1(0.28%)	0(0.00%)	0(0.00%)	0.4251
**Unsolicited AR**	**0(0.00%)**	**1(0.28%)**	**0(0.00%)**	**1(0.28%)**	**0(0.00%)**	**0(0.00%)**	**0(0.00%)**	**0.5446**
Local AR	0(0.00%)	1(0.28%)	0(0.00%)	1(0.28%)	0(0.00%)	0(0.00%)	0(0.00%)	0.5446
Discoloration	0(0.00%)	0(0.00%)	0(0.00%)	1(0.28%)	0(0.00%)	0(0.00%)	0(0.00%)	0.4229
Hypoesthesia	0(0.00%)	1(0.28%)	0(0.00%)	0(0.00%)	0(0.00%)	0(0.00%)	0(0.00%)	0.4251

Results are represented as n (%), the number and incidence of each AR. The P value is calculated using Fisher’s exact test.

The safety results for subgroup analyses by age were shown in **Table S3**. The overall incidence of adverse reactions was highest in participants aged 3-5 years (14.64%), which was followed by 12-17 years (6.22%) and 6-11 years (4.75%). There were some statistically significant differences among the three age groups for some specific adverse reactions, including rash and pain at the injection site, fever, fatigue, diarrhea, and cough (**Table S3**).

## 4 Discussion

In this phase IV, randomized, double-blind clinical trial in healthy children and adolescents aged 3-17 years, we found equivalent immune responses after CoronaVac vaccination among all comparative groups of different lots or workshops, where the 95%CIs of GMT ratios were all within the equivalence criterion of [0.67, 1.5]. These results were consistent with our previous phase IV study in healthy adults aged 26-45 years, where lot-to-lot consistency was also observed for three lots of CoronaVac on a commercial scale, with GMT ratios of 1.16 (95%CI: 1.01, 1.32), 1.15 (95%CI: 1.01, 1.32), and 0.99 (95%CI: 0.87, 1.14) for each pair of lots, respectively (13). These lot-to-lot consistency data on two types of populations provided evidence to support stable manufacturing of commercial-scale CoronaVac produced by Sinovac, as well as good stability and consistency of the two newly established workshops.

In this trial, the observed GMT of neutralizing antibody was 126.42 for all participants at 28 days after full course of vaccination, ranging from 118.78 to 134.97 among seven lots; and the immunogenicity level in children and adolescents were much higher than that in adults aged 26-45 years in our previous lot-to-lot consistency study, of which the observed GMT were 68.4 ranging from 65.0 to 75.2 among three lots (13). A similar trend was also observed in our previous phase II clinical trials on CoronaVac, where the GMTs at 28 days after full course of vaccination were 142.2, 44.1 and 54.9 for children and adolescents aged 3-17 year, adults aged 18-59 years, and adults aged ≥60 years (12, 14, 15). In subgroup analyses of this trial, an increased GMT level was also found with age decreasing, where the highest GMT was in participants aged 3-5 years (240.86), followed by participants aged 6-11 years (155.12) and 12-17 years (85.67). Although the underlying physiologic mechanisms remain incompletely understood, cumulative evidence suggests distinct SARS-CoV-2 antibody responses in children and adults (17, 18), and the early robust innate immune response and trained immunity may play an important role (19). Several studies also found that children tended to have higher levels of antibody responses after SARS-CoV-2 infection compared to adults (20, 21). In terms of seroconversion rate at 28 days after full course of vaccination, it was as high as 99.86% for all participants in this trial, which was in similar level to the seroconversion rate in all age populations in our previous clinical trials (12–15). In addition, some studies on vaccine effectiveness of CoronaVac in pediatric populations found two doses of primary immunization could provide effective protection against COVID-19 (22–24), which complement the immunogenicity results of this study. Collectively, despite GMT levels varied in different age population after vaccination in our studies, similar and high seroconversion rate were observed in the pediatric and adult populations. These immunogenicity data indicated that CoronaVac vaccination could induce a good immune response, especially for children and adolescents.

In addition, the incidences of adverse reactions were similar among seven lots in this trial, with no statistical difference. CoronaVac showed a well-tolerated safety profile in children and adolescents that most adverse reactions were mild (grade 1) in severity, with no SAE and AESI reported. These findings were consistent with our previous phase I/II study as well as the following homologous booster study in children and adolescents (12, 25), and the most frequently reported local and systemic adverse reactions were injection site pain, fever and cough in all studies. We also observed a higher incidence of adverse reactions in children aged 3-5 years in this study, while in our previous phase I/II clinical trials in children the highest incidence was observed in children aged 12-17 years (38%), followed by that in children aged 3-5 years (27%) and 6-11 years (22%) (12). This difference could be explained by different sample sizes and study seasons between these two studies. Overall, commercial-scale CoronaVac is safe and stably manufactured for vaccination campaign of pediatric population.

There were several limitations in this study. Firstly, given that when the study started, large vaccination campaigns had been implemented and good safety profiles had been observed for CoronaVac in real world settings, the study period was only set up to 28 days after two-dose vaccination. The relatively short follow-up period could not evaluate a long-term immunogenicity and safety of CoronaVac among children and adolescents. Secondly, only neutralizing antibody titers against ancestral strain was tested for the immunogenicity assessment. Due to continuous genetic mutations of SARS-CoV-2 and changes in its characteristics, we need to assess the lot-to-lot consistency of CoronaVac against SARS-CoV-2 variants. Thirdly, this study was conducted during the period of China’s dynamic Zero-COVID policy implementation, and very few COVID-19 cases occurred. This study background is different form the read word settings in other countries, but it can also avoid the confusion causing by SARS-CoV-2 natural infections.

In conclusion, among healthy children and adolescents aged 3-17 years, two doses of CoronaVac are well-tolerated and can induce a good and similar immune response among seven lots from three workshops. Lot-to-lot consistency evidence indicates stable manufacturing of commercial-scale CoronaVac by Sinovac.

## Data availability statement

The original contributions presented in the study are included in the article/**Supplementary Material**. Further inquiries can be directed to the corresponding authors.

## Ethics statement

The studies involving humans were approved by Shaanxi Provincial Center for Disease Control and Prevention, China. The studies were conducted in accordance with the local legislation and institutional requirements. Written informed consent for participation in this study was provided by the participants’ legal guardians/next of kin.

## Author contributions

WH: Investigation, Methodology, Supervision, Validation, Writing – review & editing. XLu: Investigation, Methodology, Supervision, Validation, Writing – review & editing. XL: Investigation, Methodology, Software, Supervision, Validation, Writing – review & editing. DZ: Investigation, Supervision, Writing – review & editing. SL: Validation, Writing – original draft. XG: Investigation, Supervision, Writing – review & editing. DL: Investigation, Supervision, Writing – review & editing. JS: Investigation, Supervision, Writing – review & editing. TZ: Investigation, Writing – review & editing. XLi: Data curation, Formal analysis, Software, Writing – review & editing. YG: Methodology, Project administration, Validation, Writing – review & editing. YZ: Methodology, Project administration, Validation, Writing – review & editing. GC: Conceptualization, Funding acquisition, Resources, Writing – review & editing. SZ: Conceptualization, Writing – review & editing.
