# Supplementary material for: Immunogenicity, safety and consistency of seven lots of an inactivated COVID-19 vaccine in healthy children and adolescents: a randomized, double-blind, controlled, phase IV clinical trial
Source: Front Immunol. 2024 Jan 5;14:1320352. doi: 10.3389/fimmu.2023.1320352 (PMC10796506; doi:10.3389/fimmu.2023.1320352)
Supplement: Supplementary file 1 [file Table_1.docx]

**Immunogenicity, safety and consistency of seven lots of an inactivated COVID-19 vaccine (CoronaVac) in healthy children and adolescents: a randomized, double-blind, controlled, phase Ⅳ clinical trial**

Weijun Hu^†^, Xiaoyu Liu^†^, Xi Lu^†^, Dan Zhang, Shuo Liu, Xianjin Gu, Dan Liu, Jianwen Sun, Tiantian Zhou, Xinge Li, Yongjun Gao, Yanwei Zhao, Guoliang Cui*, Shaobai Zhang*.

^†^These authors contributed equally to this work and hare first authorship.

***Correspondence:** Guoliang Cui, cuigl@sinovac.com; Shaobai Zhang, maolyzhang@163.com

**Supplemental Materials**

**Table S1. Immunogenicity of neutralizing antibody before and after two-dose vaccination among different age groups (PPS)**

|  | **3-5 years**  (N=290) | **6-11 years**  (N=887) | **12-17 years**  (N=947) | **Total**  (N=2,124) | **P** |
| --- | --- | --- | --- | --- | --- |
| **Before vaccination** |  |  |  |  |  |
| **Seropositive** |  |  |  |  | 1.0000 |
| n (%) | 0(0.00) | 1(0.11) | 2(0.21) | 3(0.14) |  |
| 95%CI | NA | (0.00, 0.63) | (0.03, 0.76) | (0.03, 0.41) |  |
| **GMT** | 2.04 | 2.05 | 2.06 | 2.06 | 0.4504 |
| 95%CI | (2.01, 2.07) | (2.04, 2.07) | (2.04, 2.08) | (2.04, 2.07) |  |
| **At 28 days after vaccination** | |  |  |  |  |
| **Seropositive** |  |  |  |  |  |
| n (%) | 290(100.00) | 887(100.00) | 944(99.68) | 2,121(99.86) | 0.3648 |
| 95%CI | (98.74, 100.00) | (99.58, 100.00) | (99.08, 99.93) | (99.59, 99.97) |  |
| **Seroconversion** |  |  |  |  |  |
| n (%) | 290(100.00) | 887(100.00) | 944(99.68) | 2,121(99.86) | 0.3648 |
| 95%CI | (98.74, 100.00) | (99.58, 100.00) | (99.08, 99.93) | (99.59, 99.97) |  |
| **GMT** | 240.86 | 155.12 | 85.67 | 126.42 | <0.0001 |
| 95%CI | (222.78, 260.42) | (147.88, 162.71) | (81.06, 90.55) | (121.82, 131.19) |  |
| **GMI** | 118.15 | 75.53 | 41.52 | 61.49 | <0.0001 |
| 95%CI | (109.38, 127.63) | (71.91, 79.32) | (39.24, 43.94) | (59.21, 63.85) |  |

Seropositive indicates the GMT level ≥1:8. Seroconversion indicates the GMT changes from <1:8 to ≥1:8 after vaccination, or at least 4-fold increase after vaccination if baseline GMT is ≥1:8.

**Table S2. Incidence of adverse reactions with 28 days after the first and second dose of vaccination (SS1 and SS2)**

|  | **SS1 (N=2,513)** | **SS2 (N=2,387)** | **P** |
| --- | --- | --- | --- |
| **Solicited AR** | 143(5.69%) | 40(1.68%) | <0.0001 |
| Local AR | 46(1.83%) | 22(0.92%) | 0.0070 |
| Erythema | 7(0.28%) | 3(0.13%) | 0.3449 |
| Rash | 7(0.28%) | 3(0.13%) | 0.3449 |
| Pain | 33(1.31%) | 18(0.75%) | 0.0664 |
| Induration | 7(0.28%) | 5(0.21%) | 0.7750 |
| Swelling | 7(0.28%) | 5(0.21%) | 0.7750 |
| Pruritus | 6(0.24%) | 0(0.00%) | 0.0315 |
| Systemic AR | 112(4.46%) | 20(0.84%) | <0.0001 |
| Fever | 41(1.63%) | 7(0.29%) | <0.0001 |
| Fatigue | 14(0.56%) | 6(0.25%) | 0.1170 |
| Nausea | 6(0.24%) | 2(0.08%) | 0.2900 |
| Diarrhea | 10(0.40%) | 1(0.04%) | 0.0119 |
| Vomiting | 8(0.32%) | 1(0.04%) | 0.0394 |
| Decreased appetite | 3(0.12%) | 2(0.08%) | 1.0000 |
| Headache | 6(0.24%) | 1(0.04%) | 0.1256 |
| Myalgia | 4(0.16%) | 1(0.04%) | 0.3756 |
| Cough | 44(1.75%) | 8(0.34%) | <0.0001 |
| Hypersensitivity | 1(0.04%) | 0(0.00%) | 1.0000 |
| Mucocutaneous disorder | 1(0.04%) | 0(0.00%) | 1.0000 |
| **Unsolicited AR** | 2(0.08%) | 0(0.00%) | 0.5002 |
| Local AR | 2(0.08%) | 0(0.00%) | 0.5002 |
| Discoloration | 1(0.04%) | 0(0.00%) | 1.0000 |
| Hypoesthesia | 1(0.04%) | 0(0.00%) | 1.0000 |

Results are represented as n (%), the number and incidence of each AR. The P value is calculated using Fisher’s exact test.

**Table S3. Overall incidence of adverse reactions after two-dose vaccination by age group (SS, 0-56 days)**

|  | **3-5 years (N=362)** | **6-11 years (N=1,074)** | **12-17 years (N=1,077)** | **All ages (N=2,513)** | **P** |
| --- | --- | --- | --- | --- | --- |
| **Overall AR** | **53(14.64%)** | **51(4.75%)** | **67(6.22%)** | **171(6.80%)** | **<0.0001** |
| **Solicited AR** | **53(14.64%)** | **51(4.75%)** | **66(6.13%)** | **170(6.76%)** | **<0.0001** |
| Local AR | 10(2.76%) | 16(1.49%) | 33(3.06%) | 59(2.35%) | 0.0466 |
| Erythema | 0(0.00%) | 4(0.37%) | 4(0.37%) | 8(0.32%) | 0.5090 |
| Rash | 4(1.10%) | 3(0.28%) | 2(0.19%) | 9(0.36%) | 0.0343 |
| Pain | 4(1.10%) | 11(1.02%) | 28(2.60%) | 43(1.71%) | 0.0119 |
| Induration | 1(0.28%) | 4(0.37%) | 6(0.56%) | 11(0.44%) | 0.7140 |
| Swelling | 1(0.28%) | 4(0.37%) | 6(0.56%) | 11(0.44%) | 0.7140 |
| Pruritus | 2(0.55%) | 0(0.00%) | 4(0.37%) | 6(0.24%) | 0.0880 |
| Systemic AR | 48(13.26%) | 38(3.54%) | 41(3.81%) | 127(5.05%) | <0.0001 |
| Fever | 18(4.97%) | 16(1.49%) | 12(1.11%) | 46(1.83%) | <0.0001 |
| Fatigue | 4(1.10%) | 2(0.19%) | 12(1.11%) | 18(0.72%) | 0.0246 |
| Nausea | 0(0.00%) | 4(0.37%) | 3(0.28%) | 7(0.28%) | 0.5086 |
| Diarrhea | 0(0.00%) | 0(0.00%) | 11(1.02%) | 11(0.44%) | 0.0006 |
| Vomiting | 1(0.28%) | 4(0.37%) | 4(0.37%) | 9(0.36%) | 0.9610 |
| Decreased appetite | 1(0.28%) | 1(0.09%) | 2(0.19%) | 4(0.16%) | 0.7208 |
| Headache | 1(0.28%) | 3(0.28%) | 3(0.28%) | 7(0.28%) | 1.0000 |
| Myalgia | 2(0.55%) | 0(0.00%) | 3(0.28%) | 5(0.20%) | 0.0924 |
| Cough | 34(9.39%) | 16(1.49%) | 2(0.19%) | 52(2.07%) | <0.0001 |
| Hypersensitivity | 1(0.28%) | 0(0.00%) | 0(0.00%) | 1(0.04%) | 0.0512 |
| Mucocutaneous disorder | 0(0.00%) | 0(0.00%) | 1(0.09%) | 1(0.04%) | 0.5133 |
| **Unsolicited AR** | 0(0.00%) | 0(0.00%) | 2(0.19%) | 2(0.08%) | 0.2633 |
| Local AR | 0(0.00%) | 0(0.00%) | 2(0.19%) | 2(0.08%) | 0.2633 |
| Discoloration | 0(0.00%) | 0(0.00%) | 1(0.09%) | 1(0.04%) | 0.5133 |
| Hypoesthesia | 0(0.00%) | 0(0.00%) | 1(0.09%) | 1(0.04%) | 0.5133 |

Results are represented as n (%), the number and incidence of each AR. The P value is calculated using Fisher’s exact test.
